# Supplementary material for: VEGF amplifies transcription through ETS1 acetylation to enable angiogenesis
Source: Nat Commun. 2017 Aug 29;8:383. doi: 10.1038/s41467-017-00405-x (PMC5575285; doi:10.1038/s41467-017-00405-x)
Supplement: Supplementary file 1 — Supplementary Information [file 41467_2017_405_MOESM1_ESM.pdf]

File Name: Supplementary Information
Description: Supplementary Figures and Supplementary Tables.

File Name: Peer Review File
Description:

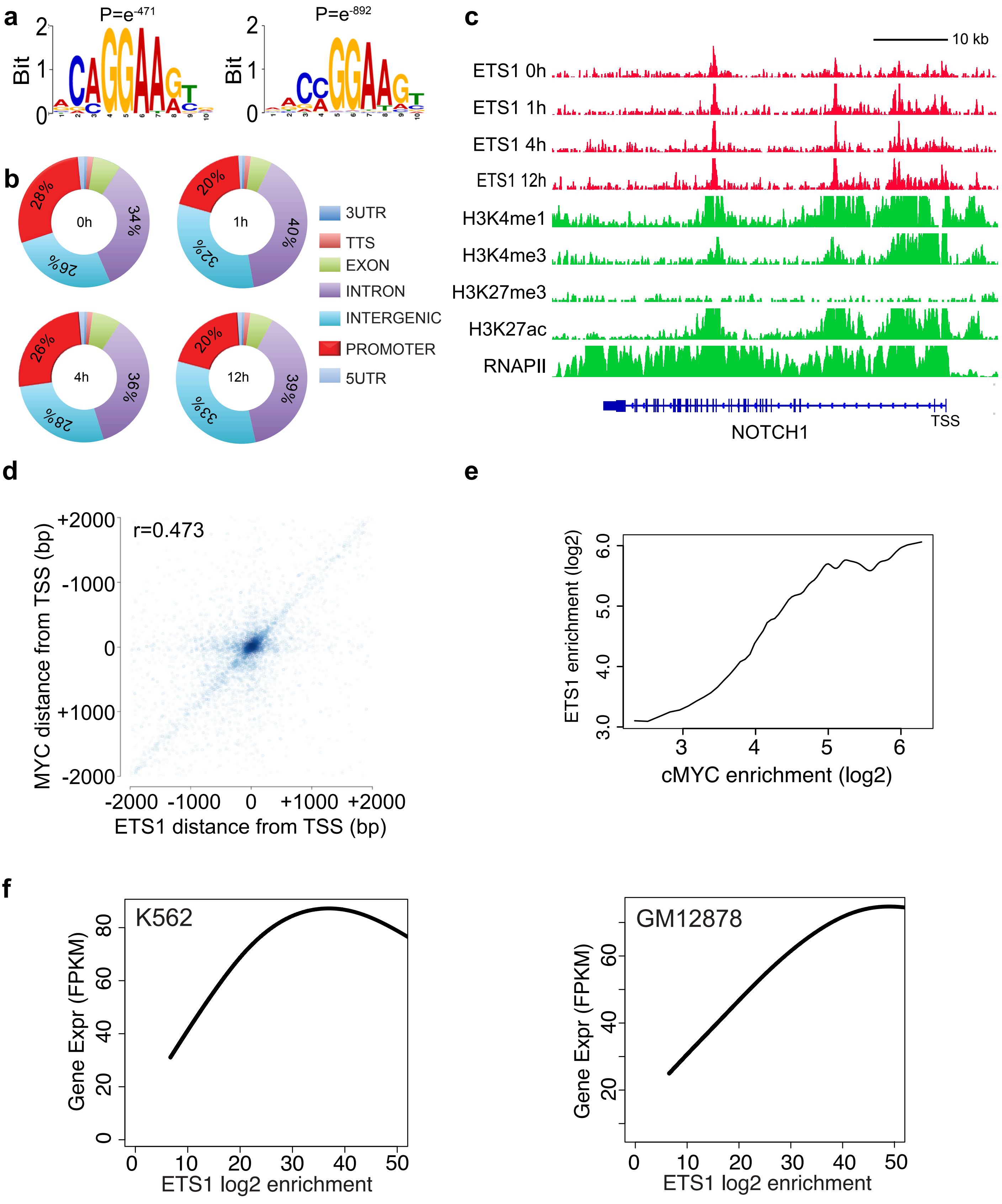

Supplementary. Fig. 1. ETS1 preferentially occupied promoters and correlated with transcription.

a. The ETS motif was highly enriched in HUVEC ETS1 ChIP-seq peaks.

b. ETS1 bound regions at 0, 1, 4, and 12 hours with respect to genome annotations.

c. Genome browser view of *NOTCH1* genomic region occupancy by ETS1 and modified histones.

d. Relationship of MYC and ETS1 ChIP-seq peaks at gene promoters. Most peaks for ETS1 and MYC overlapped in promoters.

e. Correlation between MYC enrichment and ETS1 enrichment at gene promoters.

f. Correlation of gene expression to ETS1 promoter occupancy in K562 or GM12878 cell lines.

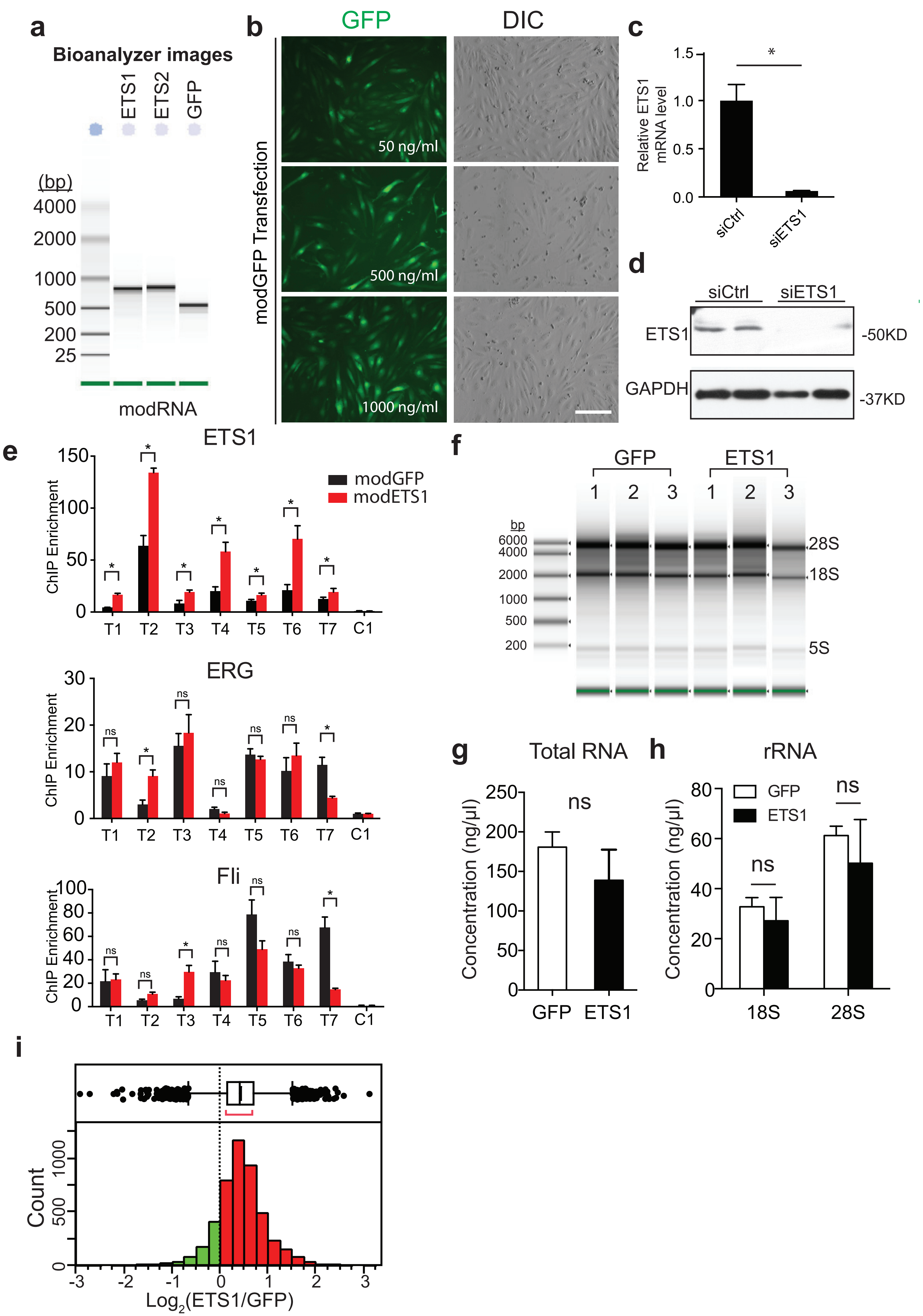
a
Bioanalyzer images
ETS1
ETS2
GFP
(bp)
4000
2000
1000
500
200
25
modRNA
b
GFP
DIC
modGFP Transfection
50 ng/ml
500 ng/ml
1000 ng/ml
c
Relative ETS1 mRNA level
1.5
1.0
0.5
0.0
*
siCtrl
siETS1
d
siCtrl
siETS1
ETS1
-50KD
GAPDH
-37KD
e
ETS1
ChIP Enrichment
modGFP
modETS1
T1
T2
T3
T4
T5
T6
T7
C1
ERG
ns
*
Fli
f
GFP
ETS1
1
2
3
bp
6000
4000
2000
1000
500
200
28S
18S
5S
g
Total RNA
Concentration (ng/μl)
ns
GFP
ETS1
h
rRNA
GFP
ETS1
18S
28S
i
Count
Log2(ETS1/GFP)

Supplementary Fig. 2. Increasing ETS1 using modified mRNA (modRNA) transfection increased mRNA abundance in HUVEC cells.

a. Bioanalyzer evaluation of ETS1, ETS2, and GFP modRNAs, synthesized by in vitro transcription.

b. Epifluorescent and phase images of HUVEC transfected by different doses of modRNA. Transfection was highly efficient, non-toxic, and peak translated protein was observed within several hours. Bar = 200 μm.

c-d. ETS1 depletion in HUVEC by siRNA at the mRNA (c) and protein (d) levels. Student's t-test: *, $P<0.05$. n=3. Error bars: s.e.m.

e. ChIP-qPCR measurement of ETS1, ERG, and FLI chromatin occupancy. Seven sites (T1-T7) and a negative control site (C1) were selected by their occupancy by ERG and FLI in HUVEC and by ETS1 in ETS1 modRNA-treated HUVEC. ChIP-qPCR was performed in HUVEC treated with modRNA encoding ETS1 or GFP. Student's t-test: *, $P<0.05$. ns, not significant. n=3. Error bars: s.e.m.

f. Tape station analysis of RNA. Total RNA from 2x105 cells were extracted with miRNA Easy Mini Kit and applied to Agilent 2200 Tape Station RNA ChiP

g. Quantification of total RNA by Tape Station. ns, not significant by Student's t-test. n=3. Error bars: s.d.

h. Quantification of rRNA by Tape Station. ns, not significant by Student's t-test. n=3. Error bars: s.d.

i. RNA-seq data were used to calculate fold-change of gene expression by ETS1 overexpression. Genes with FPKM < 2 in either group were excluded. The expression of 84% of genes increased (log2 FC > 0) and 16% decreased.

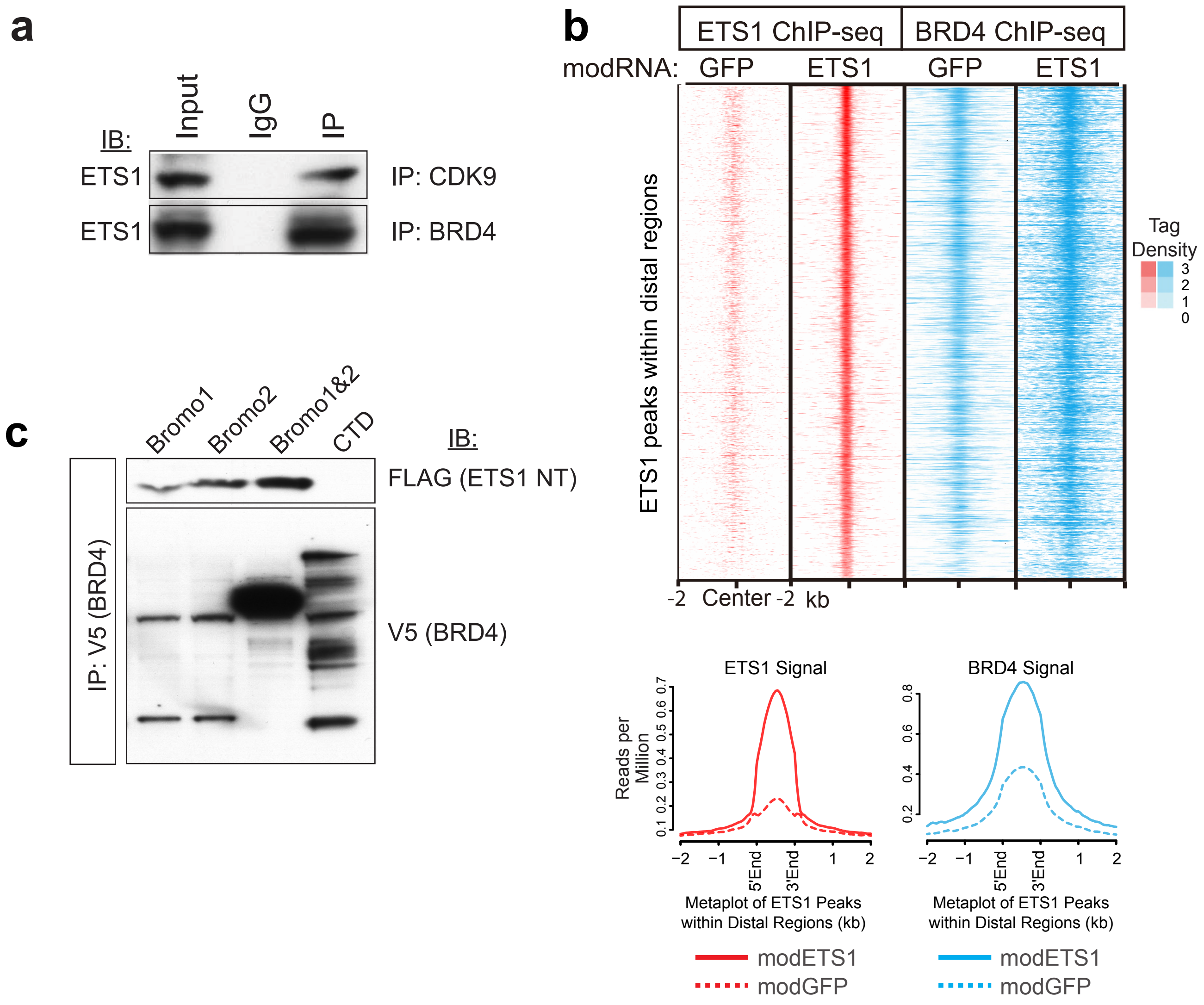

Supplementary Fig. 3. ETS1 binds and recruits BRD4 to chromatin.

a. BRD4 or CDK9 co-immunoprecipitated ETS1 from HUVECs. HUVEC nuclear extracts were incubated with BRD4 or CDK9 antibody. Immunoprecipitates were probed with ETS1 antibody.

b. BRD4 and ETS1 co-occupied chromatin in distal regions, and ETS1 modRNA over-expression stimulated BRD4 co-occupancy. Top panel, tag heat map of distal ETS1 regions. Bottom panels, aggregation plots for ETS1 or BRD4 signal in distal ETS1 regions of HUVECs treated with modGFP or modETS1.

c. BRD4 co-immunoprecipitated ETS1 NT domain. 293T cells were transfected with expression constructs encoding FLAG-ETS1-NT domain and the indicated V5-tagged BRD4 domains. V5 immunoprecipitates were probed for ETS1-NT co-precipitation using FLAG antibody.

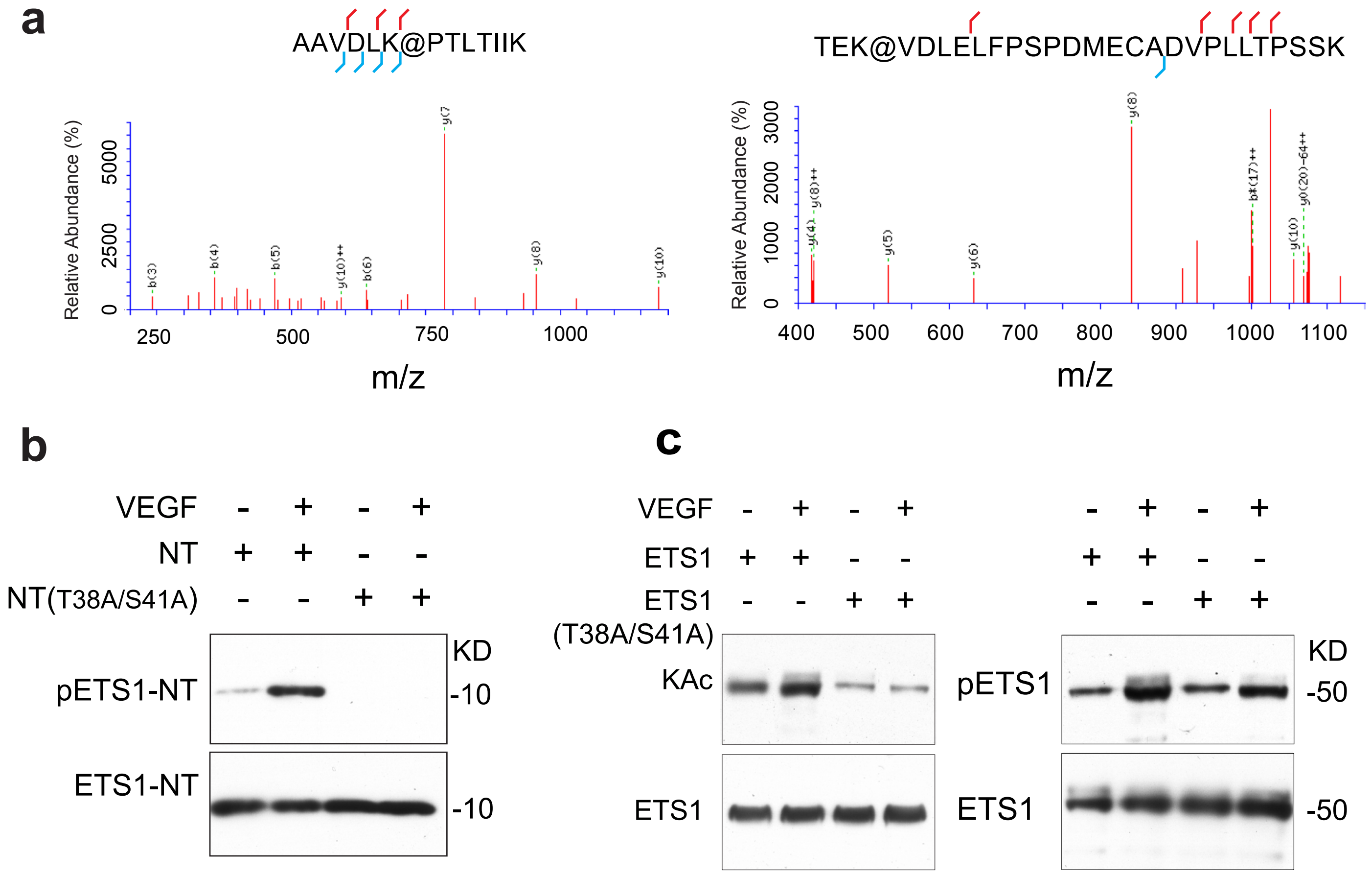

Supplementary Fig. 4. VEGF stimulated ETS1 phosphorylation and acetylation.

a. Mass spectrum of the two major acetylated ETS1 fragments. ETS1 was immunoprecipitated from HUVECs in EGM-2 media (which contains VEGF) and analyzed by mass spectroscopy. These fragments map to the NT domain of ETS1. @ indicates the detected acetylation site. Lines in the fragment sequence indicate ionizated fragments detected by LTQ Orbitrap.

a. VEGF stimulated ETS1 phosphorylation at T38 and S41. HUVECs were transfected with expression constructs encoding ETS1 NT domain or a mutant lacking ERK phosphorylation sites (T38A/S41A). ETS1 pT38 antibody (pETS1) was used to detect ERK-phosphorylated ETS1.

b. VEGF-stimulated ETS1 acetylation requires ERK T38 and S41 phosphorylation sites. (Left panel): Wild-type ETS1 was acetylated at baseline and this increased with VEGF treatment. T38A/S41A ETS1 had less baseline ETS1 and this did not increase with VEGF treatment. (Right panel): Wild-type ETS1 phosphorylation was increased by VEGF. T38A/S41A ETS1 phosphorylation also increased with VEGF, but to a lesser degree than wild-type. This suggests that T38 and S41 are the major but not exclusive sites of ETS1 that are phosphorylated downstream of VEGF.

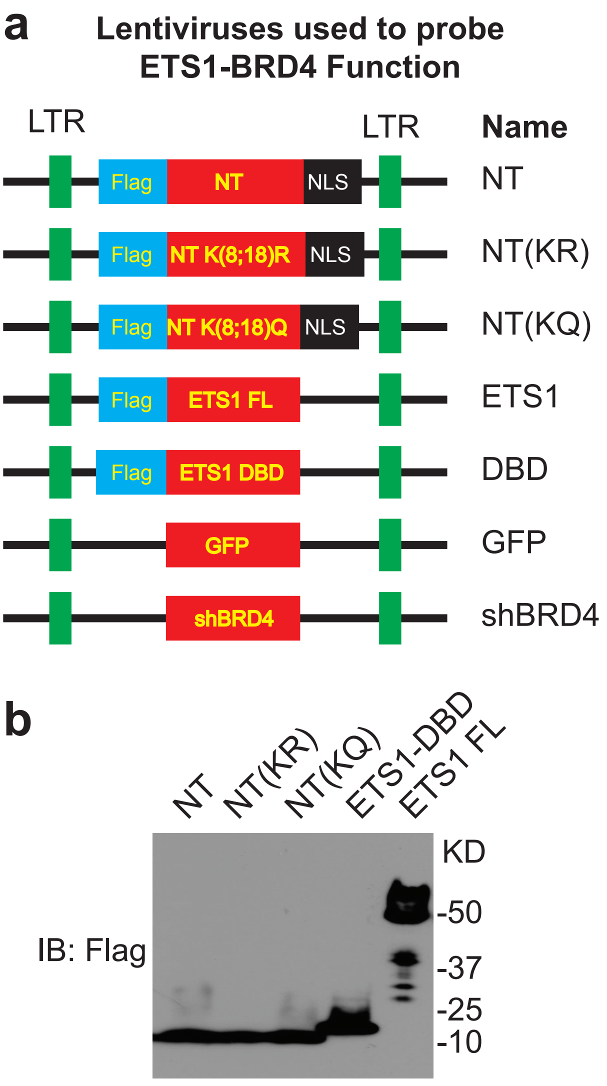

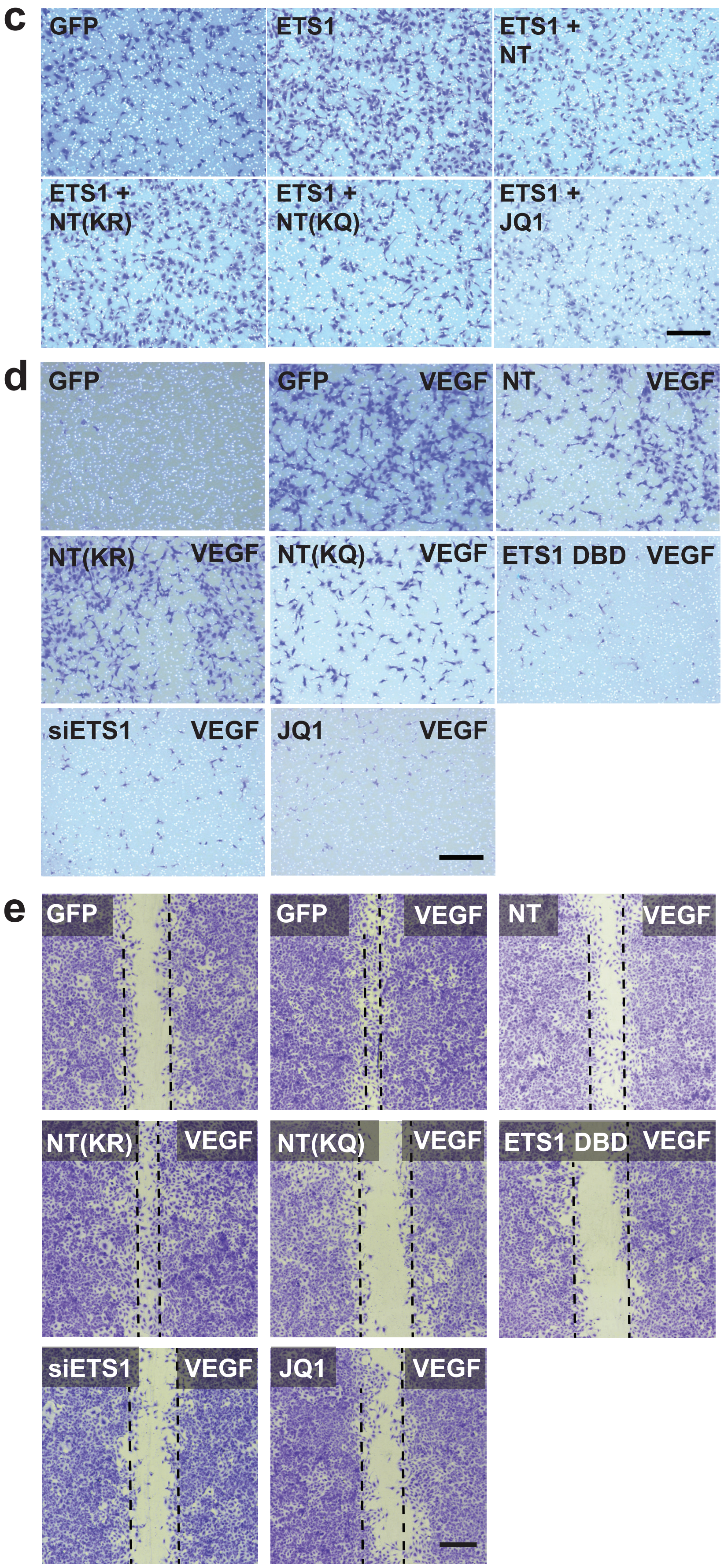

Supplementary Fig. 5. Lentiviral constructs used in this study.

a. Schematic of lentiviral constructs (not drawn to scale). Lentiviruses expressed ETS1 NT domain, NT domain mutations, full length ETS1, ETS1 DNA-binding domain (DBD), GFP, or shRNAs against ETS1 or BRD4.

b. Immunoblot showing lentiviral expression of indicated proteins.

c. Representative images of transwell assay of ETS1-driven HUVEC cell migration. The cell treatment is as indicated. Bar=250 μm.

d. Representative images of transwell assay of VEGF-driven HUVEC cell migration. The cell treatment is as indicated. Bar=250 μm.

e. Representative images of VEGF-driven HUVEC wound healing. The cell treatment is as indicated. Bar=500 μm.

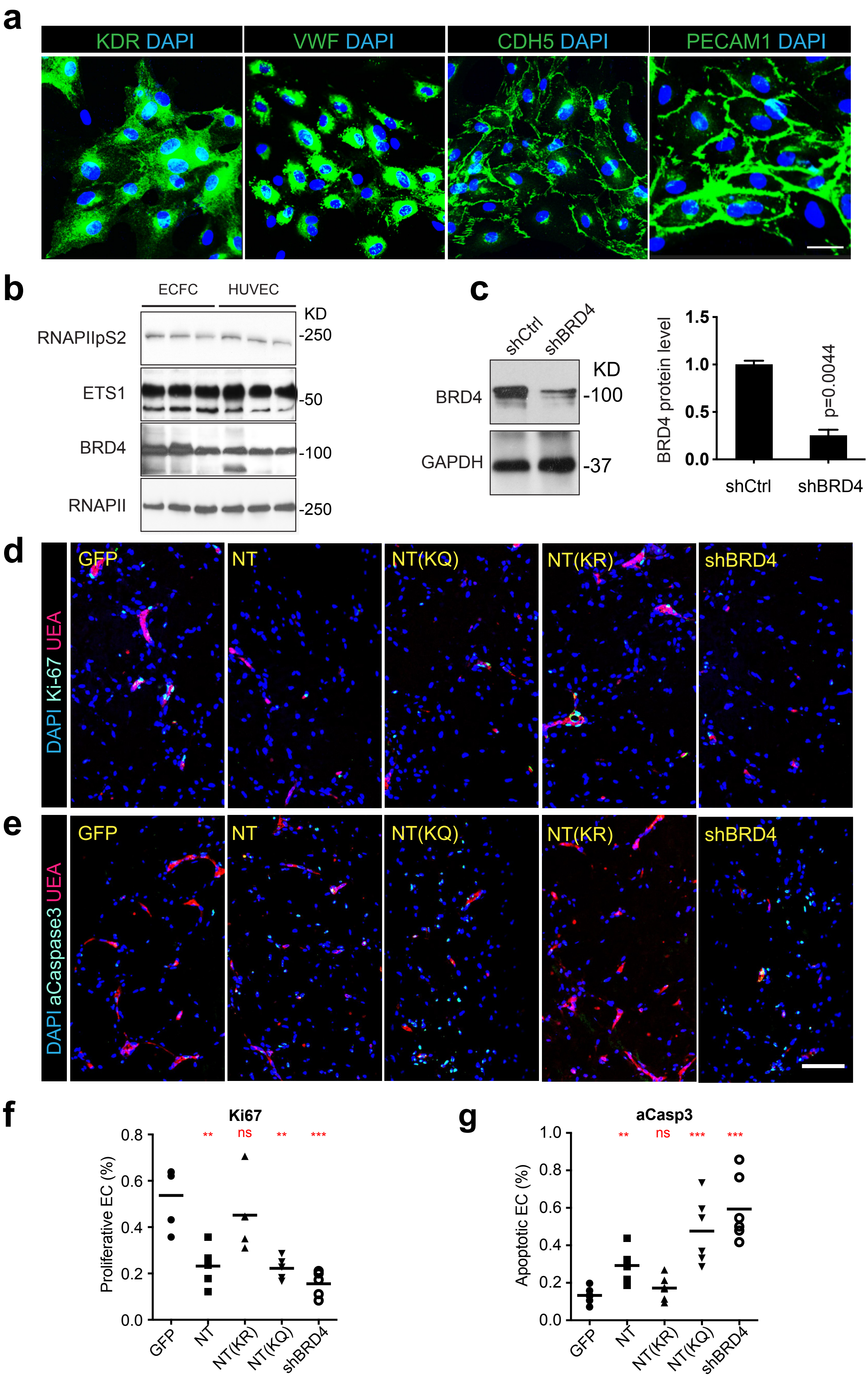

Supplementary Fig. 6. Vessel assembly in matrigel plug assay required ETS-BRD4 interaction and BRD4 activity.

a. ECFCs uniformly expressed the enothelial markers of KDR, VWF, CDH5 and PECAM1. Bar = 100 µm.

b. ECFCs and HUVECs expressed similar levels of ETS1, BRD4, RNAPII, and RNAPIIpS2.

c. Lentivirus expressing shBRD4 reduced BRD4 protein. Student's t-test, n=3. Error bars: s.d.

d-e. Representative immunofluoresent staining of EC proliferation and apoptosis in matrigel plugs. Matrigel plug sections were stained for human ECs (UEA) and proliferation (Ki67) or apoptosis (activated Caspase 3) markers. Bar = 100 µm.

f-g. Quantitative analysis of d-e. Student's t-test versus GFP: **, p<0.01; ***, P<0.001. ns, not significant.

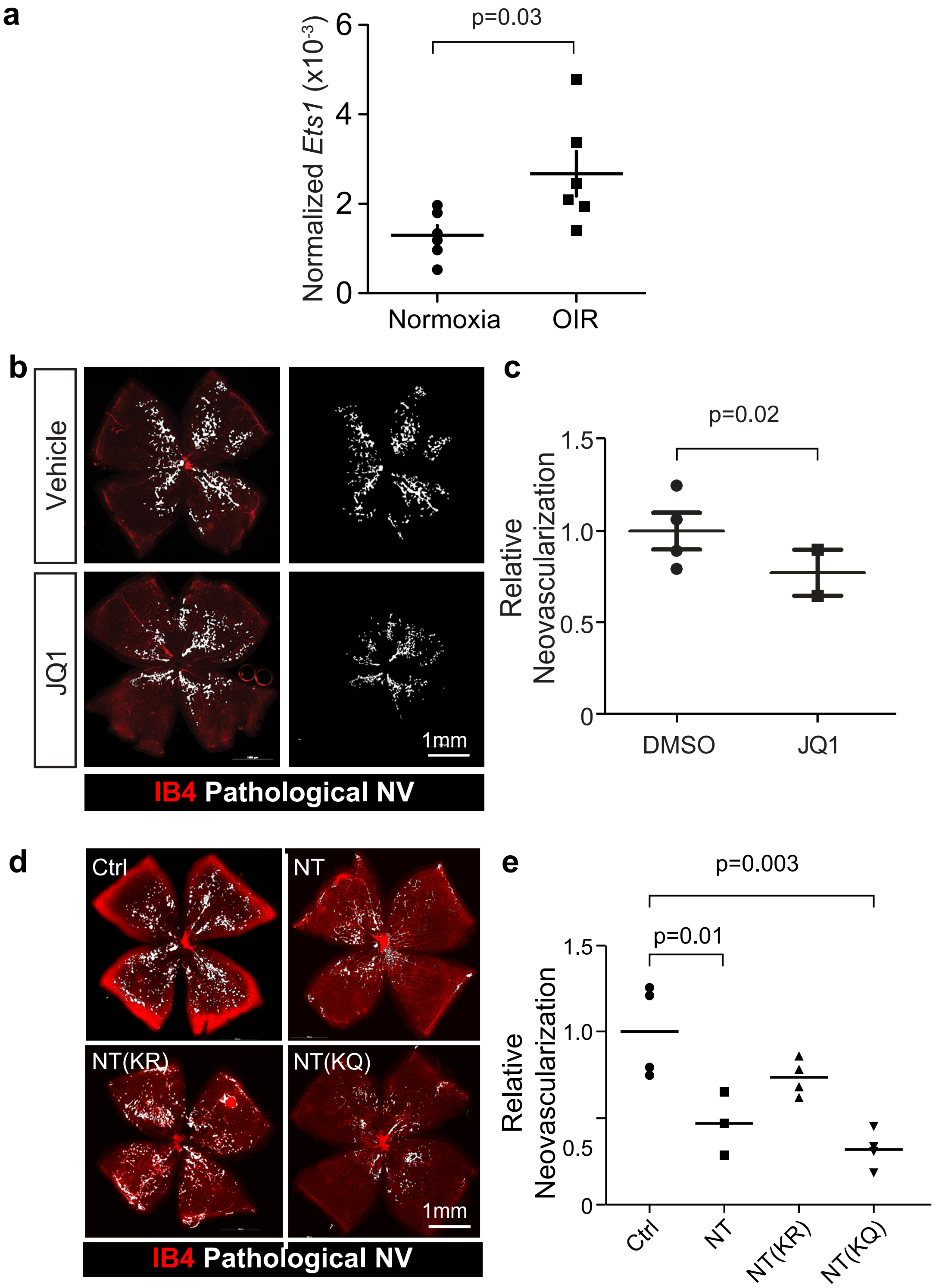

Supplementary. Fig. 7. ETS1-BRD4 promote neovascularization in oxygen-induced retinopathy.

a. *Ets1* transcript level in control and OIR retina at postnatal day 17, relative to *Gapdh*. n=6. Student's t-test.

b. Representative images of OIR retinas from mice treated with vehicle or JQ1. Retinas were stained with isolectin IB4 to visualize vessels. Pathological neovascularization (NV), identified using the SWIFT NV ImageJ plug-in, is colored white. Bar = 1 mm.

c. Quantification of neovascularization in OIR retinas from control or JQ1-treated mice. Student's t-test. n=4.

d. Representative images of OIR retinas from mice treated with the indicated lentivirus, injected intravitreally into the retina. Bar = 1 mm.

e. Quantification of neovascularization in OIR retinas from lentivirus-treated mice. NT and NT(KQ) but not NT(KR) lentiviruses reduced OIR retinopathy. Student's t-test. n=4.

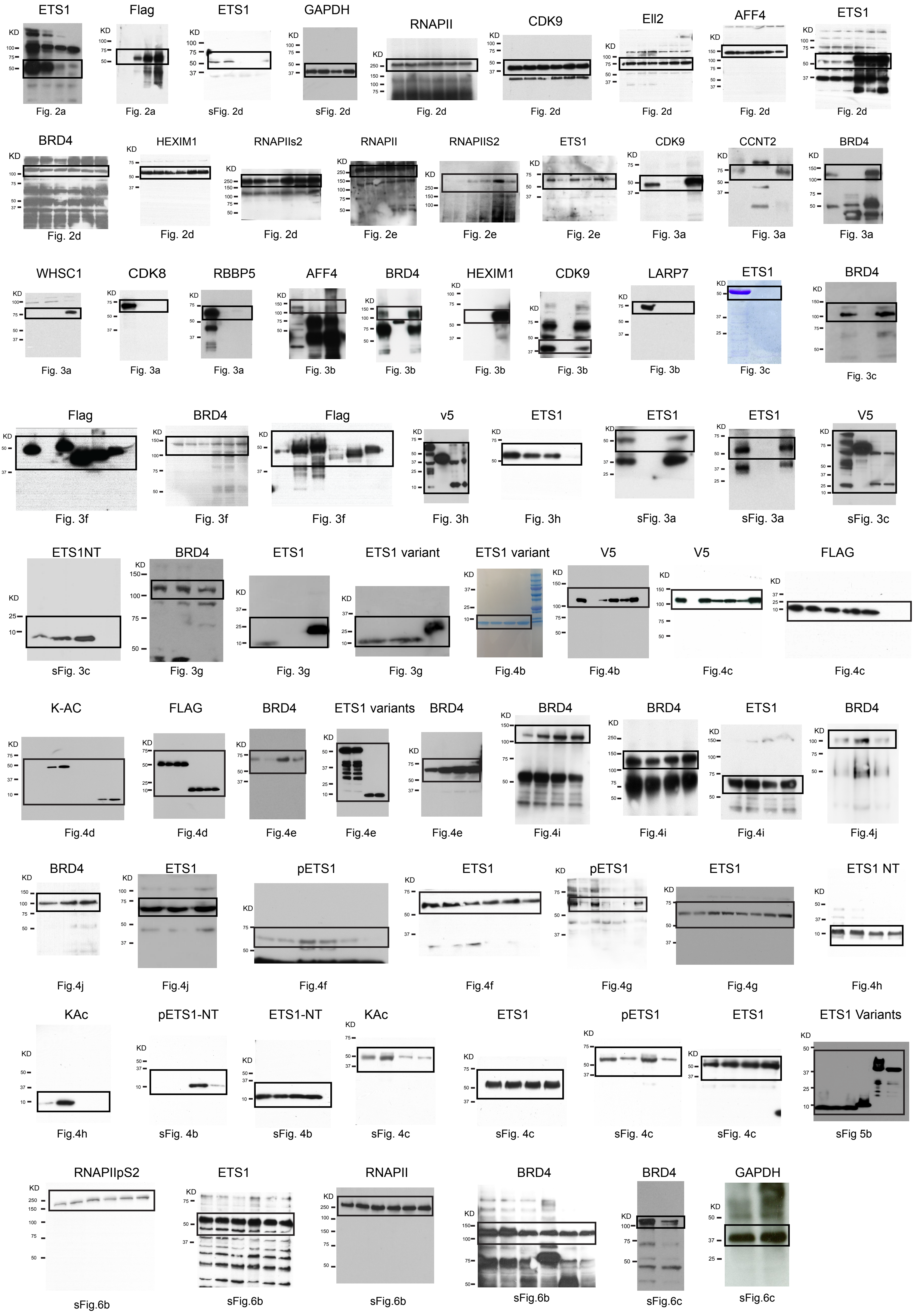
ETS1 Fig. 2a
Flag Fig. 2a
ETS1 sFig. 2d
GAPDH sFig. 2d
RNAPII Fig. 2d
CDK9 Fig. 2d
Ell2 Fig. 2d
AFF4 Fig. 2d
ETS1 Fig. 2d
BRD4 Fig. 2d
HEXIM1 Fig. 2d
RNAPIIs2 Fig. 2d
RNAPII Fig. 2e
RNAPIIS2 Fig. 2e
ETS1 Fig. 2e
CDK9 Fig. 3a
CCNT2 Fig. 3a
BRD4 Fig. 3a
WHSC1 Fig. 3a
CDK8 Fig. 3a
RBBP5 Fig. 3a
AFF4 Fig. 3b
BRD4 Fig. 3b
HEXIM1 Fig. 3b
CDK9 Fig. 3b
LARP7 Fig. 3b
ETS1 Fig. 3c
BRD4 Fig. 3c
Flag Fig. 3f
BRD4 Fig. 3f
Flag Fig. 3f
v5 Fig. 3h
ETS1 Fig. 3h
ETS1 sFig. 3a
ETS1 sFig. 3a
V5 sFig. 3c
ETS1NT sFig. 3c
BRD4 Fig. 3g
ETS1 Fig. 3g
ETS1 variant Fig. 3g
ETS1 variant Fig.4b
V5 Fig.4b
V5 Fig.4c
FLAG Fig.4c
K-AC Fig.4d
FLAG Fig.4d
BRD4 Fig.4e
ETS1 variants Fig.4e
BRD4 Fig.4e
BRD4 Fig.4i
BRD4 Fig.4i
ETS1 Fig.4i
BRD4 Fig.4j
BRD4 Fig.4j
ETS1 Fig.4j
pETS1 Fig.4f
ETS1 Fig.4f
pETS1 Fig.4g
ETS1 Fig.4g
ETS1 NT Fig.4h
KAc Fig.4h
pETS1-NT sFig. 4b
ETS1-NT sFig. 4b
KAc sFig. 4c
ETS1 sFig. 4c
pETS1 sFig. 4c
ETS1 sFig. 4c
ETS1 Variants sFig 5b
RNAPIIpS2 sFig.6b
ETS1 sFig.6b
RNAPII sFig.6b
BRD4 sFig.6b
BRD4 sFig.6c
GAPDH sFig.6c

Supplementary Fig. 8. Uncropped western blots. Original images for the western blots used in this study. Rectangles indicate approximate regions that were used in the final figures. The protein blotted is shown at the top of each image, and the figure is shown at the bottom.

Supplementary Table 1. Next Generation Datasets Used In This Study

Description	ChiP antibody	Treatment	Method	Reference	Accession Number
HUVEC	RNAPII	modGFP	ChiP-seq	This study	
HUVEC	RNAPII	modETS1	ChiP-seq	This study	
HUVEC	RNAPII	siRNA CTRL	ChiP-seq	This study	
HUVEC	RNAPII	siETS1	ChiP-seq	This study	
HUVEC	ETS1	modGFP	ChiP-seq	This study	
HUVEC	ETS1	modETS1	ChiP-seq	This study	
HUVEC	Brd4	modGFP	ChiP-seq	This study	
HUVEC	Brd4	modETS1	ChiP-seq	This study	
HUVEC	ETS1	VEGF 0h	ChiP-seq	Zhang 2013	GSE41166
HUVEC	ETS1	VEGF 1h	ChiP-seq	Zhang 2013	GSE41166
HUVEC	ETS1	VEGF 4h	ChiP-seq	Zhang 2013	GSE41166
HUVEC	ETS1	VEGF 12h	ChiP-seq	Zhang 2013	GSE41166
HUVEC	H3K27ac	VEGF 0h	ChiP-seq	Zhang 2013	GSE41166
HUVEC	H3K4me1	VEGF 0h	ChiP-seq	This study	
HUVEC	H3K4me2	VEGF 0h	ChiP-seq	This study	
HUVEC	H3K4me3	VEGF 0h	ChiP-seq	This study	
HUVEC	H3K27me3	VEGF 0h	ChiP-seq	This study	
HUVEC	H3K36me3	VEGF 0h	ChiP-seq	This study	
HUVEC	NA	modETS1	RNA-seq	This study	
HUVEC	NA	modGFP	RNA-seq	This study	
HUVEC	NA	VEGF 0h	RNA-seq	Zhang 2013	GSE41166
HUVEC	NA	VEGF 1h	RNA-seq	Zhang 2013	GSE41166
HUVEC	NA	VEGF 4h	RNA-seq	Zhang 2013	GSE41166
HUVEC	NA	VEGF 12h	RNA-seq	Zhang 2013	GSE41166
K562	ETS1	NA	ChiP-seq	ENCODE	GSM803442
K562	NA	NA	RNA-seq	ENCODE	
GM12878	ETS1	NA	ChiP-seq	ENCODE	GSM803510
GM12878	NA	NA	RNA-seq	ENCODE	

Supplementary Table 2. ETS1 K8 and K18 lysine acetylation identified by mass spectroscopy.

3x-FLAG-ETS1 and CBP were co-transfected in 293T cells. Afinity-purified 3x-FLAG-ETS1 was analyzed by mass spectroscopy. Data on selective peptides that support K8 and K18 acetylation are shown. @, acetyl-lysine. *, oxidized methionine. Periods indicate the start and end points of the peptide sequence that was identified.

ScanF	z	XCorr	ΔCorr	Ref.	Intensity	ModScore Peptide	Site 1 Score	Site 2 Score
10501	3	1.869	0.31	ETS1	1.46E+05	R.YYYDK@NIIHK.T	1000	0
14726	3	4.774	0.034	ETS1	1.18E+06	R.VPSYDSFDSEDYPAALPNHK@PK.G	1000	0
11212	2	1.604	0.808	ETS1	1.69E+06	R.LGIPK@DPR.Q	1000	0
14845	2	5.906	0.846	ETS1	7.50E+06	R.GK@LGGQDSFESIESYDSCDR.L	1000	0
15023	2	5.843	0.843	ETS1	7.50E+06	R.GK@LGGQDSFESIESYDSCDR.L	1000	0
14495	3	4.06	0.604	ETS1	1.03E+05	R.GK@LGGQDSFESIESYDSCDR.L	1000	0
15152	2	5.006	0.796	ETS1	7.50E+06	R.GK@LGGQDSFESIESYDSCDR.L	1000	0
24357	3	4.553	0.603	ETS1	15019	K.TEK@VDLELFPSPDM*ECADVPLLTPSSK.E	1000	0
22856	3	2.511	0.389	ETS1	8.74E+05	K.TEK@VDLELFPSPDM*ECADVPLLTPSSK.E	1000	0
24476	3	3.315	0.506	ETS1	15019	K.TEK@VDLELFPSPDM*ECADVPLLTPSSK.E	1000	0
23949	3	4.377	0.537	ETS1	34039.1	K.TEK@VDLELFPSPDM*ECADVPLLTPSSK.E	1000	0
22758	3	2.413	0.435	ETS1	8.74E+05	K.TEK@VDLELFPSPDM*ECADVPLLTPSSK.E	1000	0
6818	2	2.002	0.574	ETS1	1.93E+05	K.M*NYEK@LSR.G	1000	0
19855	3	4.859	0.623	ETS1	2.67E+05	K.GVDFQK@FCMNGAALCALGK.D	1000	0
11612	2	1.908	0.587	ETS1	2.16E+06	K.GTFK@DYVR.D	1000	0
18499	2	4.26	0.515	ETS1	45552.6	K.EM*M*SQALK@ATFSGFTK.E	1000	0
11048	3	4.425	0.558	ETS1	3.28E+05	K.EDVK@PYQVNGVNPAYPESR.Y	1000	0
10477	3	5.133	0.659	ETS1	9.95E+05	K.EDVK@PYQVNGVNPAYPESR.Y	1000	0
9951	2	1.788	0.533	ETS1	67653.9	K.ATFSGFTK@.E	1000	0
22047	2	2.995	0.459	ETS1	2.06E+05	K.AAVDLK@PTLTIIK.T	1000	0
19918	2	1.817	0.428	ETS1	5.94E+06	K.AAVDLK@PTLTIIK.T	1000	0
20024	2	1.642	0.402	ETS1	5.94E+06	K.AAVDLK@PTLTIIK.T	1000	0
21944	2	1.365	0.499	ETS1	2.06E+05	K.AAVDLK@PTLTIIK.T	1000	0
20141	2	1.262	0.054	ETS1	5.94E+06	K.AAVDLK@PTLTIIK.T	1000	0
18610	2	4.301	0.628	ETS1	3.76E+05	R.TGM*K@AAVDLKPTLTIIK.T	146.622	0
18857	2	3.769	0.147	ETS1	1.67E+05	K.AAVDLKPTLTIIK@TEK.V	93.154	0
20258	3	3.113	0.528	ETS1	1.30E+05	R.TGMK@AAVDLKPTLTIIK.T	62.659	0
5751	4	5.988	0.263	ETS1	3.67E+07	-.MDYK@DHDGDYKDHDIDYKDDDDKGR.T	58.699	0
20798	3	3.499	0.121	ETS1	64357.5	R.TGMK@AAVDLKPTLTIIK@TEK.V	52.403	75.738
18556	3	2.897	0.528	ETS1	5.30E+06	R.TGM*K@AAVDLKPTLTIIK.T	52.403	0
18817	3	3.296	0.115	ETS1	6.25E+05	K.AAVDLKPTLTIIK@TEK.V	46.129	0
6033	5	4.133	0.277	ETS1	1.00E+08	-.MDYK@DHDGDYKDHDIDYKDDDDKGR.T	28.986	0
5519	4	3.636	0.445	ETS1	1.87E+07	-.M*DYK@DHDGDYKDHDIDYKDDDDK.G	27.149	0
6299	4	3.361	0.268	ETS1	4.38E+06	-.MDYK@DHDGDYKDHDIDYKDDDDK.G	27.133	0
19244	3	3.012	0.065	ETS1	6.59E+05	R.TGM*K@AAVDLKPTLTIIK@TEK.V	26.105	37.549
4199	4	2.747	0.187	ETS1	33306.5	K.DHDGDYKDHDIDYK@DDDDKGR.T	22.529	0
4214	4	2.697	0.046	ETS1	14620.9	K.DHDGDYKDHDIDYK@DDDDKGR.T	22.529	0
4309	4	2.315	0.199	ETS1	4422.8	K.DHDGDYKDHDIDYKDDDDK@GR.T	21.746	0
4025	4	2.813	0.232	ETS1	3.80E+07	-.M*DYK@DHDGDYKDHDIDYKDDDDKGR.T	21.671	0
3552	5	3.192	0.233	ETS1	7.04E+07	-.M*DYK@DHDGDYKDHDIDYKDDDDKGR.T	21.606	0
5691	5	4.132	0.368	ETS1	7.04E+07	-.M*DYK@DHDGDYKDHDIDYKDDDDKGR.T	21.606	0
4091	2	2.426	0.616	ETS1	3.19E+05	-.M@*DYKDHDGDYK.D	20.985	0
6035	3	2.063	0.386	ETS1	3.65E+06	-.MDYK@DHDGDYK.D	20.625	0
22112	5	2.705	0.091	ETS1	8626.8	-.M@*DYKDHDGDYKDHDIDYKDDDDKGR.T	20.478	0
5608	4	2.634	0.341	ETS1	1.87E+07	-.M*DYK@DHDGDYKDHDIDYKDDDDK.G	20.354	0
5156	5	4.555	0.323	ETS1	7.04E+07	-.M*DYK@DHDGDYKDHDIDYKDDDDKGR.T	17.562	0
6336	3	4.887	0.329	ETS1	1.02E+06	-.MDYK@DHDGDYKDHDIDYKDDDDK.G	17.512	0
5181	5	2.704	0.141	ETS1	2.34E+06	-.M@*DYK@DHDGDYKDHDIDYKDDDDKGR.T	17.117	22.855
5144	4	2.715	0.165	ETS1	1.74E+05	K.DHDGDYKDHDIDYK@DDDDKGR.T	16.198	0.0
5288	4	3.71	0.391	ETS1	1.87E+07	-.M*DYK@DHDGDYKDHDIDYKDDDDK.G	15.586	0.0
8467	5	2.284	0.018	ETS1	59054	-.M*DYK@DHDGDYKDHDIDYKDDDDKGR.T	13.421	0.0
5652	5	2.274	0.134	ETS1	2.34E+06	-.M@*DYKDHDGDYK@DHDIDYKDDDDKGR.T	13.348	8.2

24855	5	2.692	0.188	ETS1	5.04E+03	-.M@*DYKDHDGDYKDHDIDYKDDDDKGR.T	13.078	0.0
4895	5	3.605	0.405	ETS1	7.04E+07	-.M@*DYKDHDGDYKDHDIDYKDDDDKGR.T	13.078	0.0
26217	5	2.234	0.016	ETS1	3375.1	-.M@*DYKDHDGDYKDHDIDYKDDDDKGR.T	13.078	0.0
25323	5	2.52	0.133	ETS1	5.04E+03	-.M@*DYKDHDGDYKDHDIDYKDDDDKGR.T	13.078	0.0
27013	5	3.383	0.228	ETS1	3375.1	-.M@*DYKDHDGDYKDHDIDYKDDDDKGR.T	13.078	0.0
5749	6	3.74	0.462	ETS1	5.46E+07	-.MDYK@DHDGDYKDHDIDYKDDDDKGR.T	11.777	0.0
5388	5	2.875	0.356	ETS1	7.04E+07	-.M*DYK@DHDGDYKDHDIDYKDDDDKGR.T	10.651	0.0
3827	5	3.191	0.38	ETS1	7.04E+07	-.M@*DYKDHDGDYKDHDIDYKDDDDKGR.T	9.968	0.0
5897	5	2.924	0.028	ETS1	7.04E+07	-.M@*DYKDHDGDYKDHDIDYKDDDDKGR.T	9.968	0.0
3662	5	3.829	0.277	ETS1	7.04E+07	-.M@*DYKDHDGDYKDHDIDYKDDDDKGR.T	9.968	0.0
5268	5	3.19	0.349	ETS1	7.04E+07	-.M@*DYKDHDGDYKDHDIDYKDDDDKGR.T	9.968	0.0
4226	5	2.529	0.376	ETS1	7.04E+07	-.M@*DYKDHDGDYKDHDIDYKDDDDKGR.T	9.968	0.0
5813	5	4.396	0.275	ETS1	1.00E+08	-.MDYK@DHDGDYKDHDIDYKDDDDKGR.T	9.847	0.0
24666	5	2.921	0.231	ETS1	6.36E+03	-.M@*DYKDHDGDYKDHDIDYKDDDDKGR.T	8.152	0.0
25690	5	2.475	0.254	ETS1	4.26E+03	-.M*DYK@DHDGDYKDHDIDYKDDDDKGR.T	7.824	0.0
27822	5	2.235	0.075	ETS1	3375.1	-.M*DYK@DHDGDYKDHDIDYKDDDDKGR.T	7.824	0.0
4830	5	2.74	0.216	ETS1	7.04E+07	-.M*DYK@DHDGDYKDHDIDYKDDDDKGR.T	7.824	0.0
4666	5	2.786	0.42	ETS1	7.04E+07	-.M*DYK@DHDGDYKDHDIDYKDDDDKGR.T	7.824	0.0
4369	5	2.597	0.088	ETS1	7.04E+07	-.M*DYK@DHDGDYKDHDIDYKDDDDKGR.T	7.824	0.0
4439	5	2.934	0.32	ETS1	7.04E+07	-.M@*DYKDHDGDYKDHDIDYKDDDDKGR.T	7.277	0.0
25028	5	3.44	0.392	ETS1	5.04E+03	-.M@*DYKDHDGDYKDHDIDYKDDDDKGR.T	7.277	0.0
5059	5	3.388	0.308	ETS1	7.04E+07	-.M@*DYKDHDGDYKDHDIDYKDDDDKGR.T	7.277	0.0
26594	5	2.147	0.049	ETS1	3.38E+03	-.M@*DYKDHDGDYKDHDIDYKDDDDKGR.T	7.277	0.0
6258	5	2.132	0.028	ETS1	7.04E+07	-.M@*DYKDHDGDYKDHDIDYKDDDDKGR.T	7.277	0.0
4495	5	2.679	0.328	ETS1	7.04E+07	-.M@*DYKDHDGDYKDHDIDYKDDDDKGR.T	7.277	0.0
26937	5	2.303	0.012	ETS1	3.38E+03	-.M@*DYKDHDGDYKDHDIDYKDDDDKGR.T	7.277	0.0
23928	5	2.337	0.023	ETS1	12081.3	-.M@*DYKDHDGDYKDHDIDYKDDDDKGR.T	7.277	0.0
7106	5	2.12	0.22	ETS1	1.57E+05	-.M@*DYKDHDGDYKDHDIDYKDDDDKGR.T	7.277	0.0
3939	5	3.877	0.312	ETS1	7.04E+07	-.M@*DYKDHDGDYKDHDIDYKDDDDKGR.T	7.277	0.0
24962	5	2.694	0.23	ETS1	5.04E+03	-.M@*DYKDHDGDYKDHDIDYKDDDDKGR.T	7.277	0.0
4965	5	3.261	0.151	ETS1	7.04E+07	-.M@*DYKDHDGDYKDHDIDYKDDDDKGR.T	7.277	0.0
5258	4	2.363	0.192	ETS1	1.87E+07	-.M*DYK@DHDGDYKDHDIDYKDDDDK.G	6.893	0.0
5682	5	2.273	0.184	ETS1	1.00E+08	-.MDYKDHDGDYK@DHDIDYKDDDDKGR.T	6.753	0.0
7198	5	2.608	0.287	ETS1	1.57E+05	-.M*DYK@DHDGDYKDHDIDYKDDDDKGR.T	6.32	0.0
3980	6	2.222	0.269	ETS1	3.97E+07	-.M*DYK@DHDGDYKDHDIDYKDDDDKGR.T	6.097	0.0
3896	5	3.132	0.338	ETS1	7.04E+07	-.M*DYK@DHDGDYKDHDIDYKDDDDKGR.T	5.44	0.0
14157	3	2.806	0.024	ETS1	4.32E+05	R.VPSYDSFDSEDYPAALPNHKPK@GTFK.D	5.085	0.0
4604	5	3.532	0.352	ETS1	7.04E+07	-.M@*DYKDHDGDYKDHDIDYKDDDDKGR.T	5.021	0.0
4296	5	3.217	0.306	ETS1	7.04E+07	-.M@*DYKDHDGDYKDHDIDYKDDDDKGR.T	5.021	0.0
4716	5	3.065	0.301	ETS1	7.04E+07	-.M@*DYKDHDGDYKDHDIDYKDDDDKGR.T	5.021	0.0
4770	5	3.321	0.242	ETS1	7.04E+07	-.M@*DYKDHDGDYKDHDIDYKDDDDKGR.T	5.021	0.0
6000	3	1.849	0.527	ETS1	3.65E+06	-.M@DYKDHDGDYK.D	4.16	0.0
6181	5	2.907	0.331	ETS1	1.00E+08	-.M@DYKDHDGDYKDHDIDYKDDDDKGR.T	3.841	0.0
13970	4	3.637	0.058	ETS1	4.58E+06	R.VPSYDSFDSEDYPAALPNHK@PKGTFK.D	3.696	0.0
25708	5	2.64	0.2	ETS1	4260.4	-.M*DYK@DHDGDYKDHDIDYKDDDDKGR.T	3.514	0.0
13188	4	3.456	0.04	ETS1	94388.5	R.VPSYDSFDSEDYPAALPNHKPK@GTFK.D	3.359	0.0
14042	4	3.058	0.076	ETS1	4.58E+06	R.VPSYDSFDSEDYPAALPNHKPK@GTFK.D	3.359	0.0
25240	5	3.725	0.428	ETS1	5038.9	-.M@*DYKDHDGDYKDHDIDYKDDDDKGR.T	3.213	0.0
24947	5	3.227	0.451	ETS1	5.04E+03	-.M@*DYKDHDGDYKDHDIDYKDDDDKGR.T	3.213	0.0
5922	5	4.047	0.383	ETS1	1.00E+08	-.MDYK@DHDGDYKDHDIDYKDDDDKGR.T	2.345	0.0
26515	5	2.704	0.264	ETS1	3.38E+03	-.M*DYK@DHDGDYKDHDIDYKDDDDKGR.T	2.05	0.0
3756	5	3.375	0.334	ETS1	7.04E+07	-.M@*DYKDHDGDYKDHDIDYKDDDDKGR.T	1.852	0.0
4552	5	2.844	0.256	ETS1	7.04E+07	-.M@*DYKDHDGDYKDHDIDYKDDDDKGR.T	1.852	0.0
6229	4	2.638	0.352	ETS1	4.38E+06	-.MDYKDHDGDYK@DHDIDYKDDDDK.G	1.627	0.0
3997	5	2.591	0.256	ETS1	7.04E+07	-.M@*DYKDHDGDYKDHDIDYKDDDDKGR.T	0.919	0.0

Supplementary Table 3. Oligonucleotides Used in This Study

Name	Sequence
ETS1 shRNA#1	CCGGCCGACGAGTGATGGCACTGAACTCGAGTTCAGTGCCATCACTCGTCGGTTTTTG
ETS1 shRNA#2	CCGGCCAGCTTCGACTCAGAGGACTACTCGAGTAGTCCTCTGAGTCGAAGCTGTTTTTG
Brd4 shRNA	CCGGCCTGGAGATGACATAGTCTTACTCGAGTAAGACTATGTCATCTCCAGGTTTTTG

Primers for qRTPCR

Name	sequence	
ETS1	GAGATCCTGCAGAAAGAGGATG	GGAGCGTCTGATAGGACTCTGT
BRD4	TCTACAACAAGCCTGGAGATGA	ATCTCGGTTTCTTCTGTGGGTA
EGR3	AACTCTCTTACTCCGGCTCCTT	TGAGGCTAATGATGTTGTCCTG
NR4A1	CTTCCTGGAGCTCTTCATCCTC	GCCAGGATACTGTCAATCCAGT
IL-8	CCACCCCAAATTTATCAAAGAA	CAGACAGAGCTCTCTTCCATCA
NR4A2	ATCTCTCCACAACTTCCACCAG	GCGATTGCTTAAAGGAGAAGAG
SEMA6D	AATGCATTCAATCCCATGTGTA	CACTGTGGCAGAATACAGCTTC
ADAMTS9	GCAGGGAACACAGCCTACTATC	TTTAAAACATGATCGCATCCAG
IGFBP3	AGCTCCAGGAAATGCTAGTGAG	CTGTCTTTAGCATGCCCTTTCT
MEF2C	CATAACATGCCACCATCTGC	CGTGTGTTGTGGGTATCTCG
BMP2	TTTGCACCAAGATGAACACAGC	TGTTTGTGTTTGGCTTGACGTT
BRD4	TCTACAACAAGCCTGGAGATGA	ATCTCGGTTTCTTCTGTGGGTA

modRNA

Tail_PCR_UP	TTGGACCCTCGTACAGAAGCTAATACG
Tail_PCR_Down	TTTTTTTTTTTTTTTTTTTTTTTTTTTTTTTTTTTTTTTTTTTTTTTTTTTTTTTTTTTTTTTTTTTTTTTTTTTTTTTTTTTTTTTTTTTT<br>TTTTTTTTTTTTTTTTTTTTTTTTTTCTTCCTACTCAGGCTTTATTCAAAGACCA

Primers for multiplex library construction

3' Index1	CAAGCAGAAGACGGCATACGAGATCGTGATGTGACTGGAGTTCAGACGTGTGCTCTTCCGATCT
3' Index2	CAAGCAGAAGACGGCATACGAGATACATCGGTGACTGGAGTTCAGACGTGTGCTCTTCCGATCT
3' Index3	CAAGCAGAAGACGGCATACGAGATGCCTAAGTGACTGGAGTTCAGACGTGTGCTCTTCCGATCT
3' Index4	CAAGCAGAAGACGGCATACGAGATTGGTCAGTGACTGGAGTTCAGACGTGTGCTCTTCCGATCT
3' Index5	CAAGCAGAAGACGGCATACGAGATCACTGTGTGACTGGAGTTCAGACGTGTGCTCTTCCGATCT
3' Index6	CAAGCAGAAGACGGCATACGAGATATTGGCGTGACTGGAGTTCAGACGTGTGCTCTTCCGATCT
3' Index7	CAAGCAGAAGACGGCATACGAGATGATCTGGTGACTGGAGTTCAGACGTGTGCTCTTCCGATCT
3' Index8	CAAGCAGAAGACGGCATACGAGATTCAAGTGTGACTGGAGTTCAGACGTGTGCTCTTCCGATCT
3' Index9	CAAGCAGAAGACGGCATACGAGATCTGATCGTGACTGGAGTTCAGACGTGTGCTCTTCCGATCT
3' Index10	CAAGCAGAAGACGGCATACGAGATAAGCTAGTGACTGGAGTTCAGACGTGTGCTCTTCCGATCT
5' primer	AATGATACGGCGACCACCGAGATCTACACTCTTTCCCTACACGACGCTCTTCCGATCT
